# Endoscopic ultrasound-guided hepaticogastrostomy in a patient with complex postsurgical anatomy after subtotal esophagectomy

**DOI:** 10.1055/a-2646-1668

**Published:** 2025-07-25

**Authors:** Kei Yane, Keita Seto, Koki Yoshida, Sota Hirokawa, Kotaro Morita, Yuki Ikeda, Tetsuya Sumiyoshi

**Affiliations:** 1 36737 Department of Gastroenterology, Tonan Hospital, Sapporo, Japan

In patients with complex upper gastrointestinal reconstruction, endoscopic biliary drainage is technically challenging. Recently, the usefulness of endoscopic ultrasound-guided hepaticogastrostomy (EUS-HGS) in patients with surgically altered anatomy has been increasingly demonstrated 1 2 3 4 .

A 75-year-old man with a history of distal gastrectomy with Billroth I reconstruction for a duodenal ulcer and subtotal esophagectomy for esophagogastric junction cancer presented with a recurrent tumor and obstructive jaundice. Esophageal reconstruction was performed using a free jejunal interposition graft via the presternal route. Computed tomography revealed a duodenal obstruction from the tumor recurrence, precluding transpapillary biliary drainage ( Fig. 1 ). Therefore, EUS-HGS was planned to achieve internal biliary drainage.

**Fig. 1:**
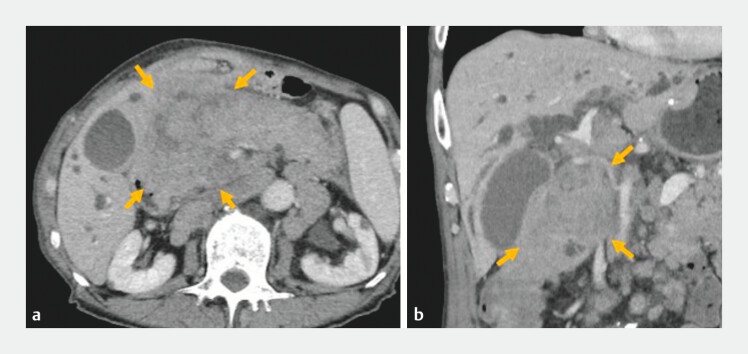
Computed tomography showing intrahepatic bile duct dilation and duodenal obstruction because of the recurrent tumor (arrows). **a** Axial image. **b** Coronal image.

A long colonoscope was used to navigate the interposed jejunal limb, allowing guidewire placement in the stomach. The colonoscope was then exchanged for a curved linear echoendoscope (EG-740UT; Fujifilm, Tokyo, Japan), and the guidewire was followed under fluoroscopic guidance to reach the stomach ( Video 1 ). From a reversed position in the upper gastric body, a dilated intrahepatic bile duct (B2) was identified ( Fig. 2 ). The puncture was performed using a 22-G fine-needle aspiration needle (Expect Slimline; Boston Scientific, Marlborough, Massachusetts, USA), and a 0.018-inch guidewire (Fielder 18; Olympus, Tokyo, Japan) was advanced. The tract was dilated using a double-lumen dilator (MEISSA; Japan Lifeline, Tokyo, Japan) and a 4-mm balloon catheter (REN; Kaneka Medics, Osaka, Japan) ( Fig. 3 ). An 8 × 120-mm covered self-expandable metal stent (Niti-S Biliary S-type; Taewoong Medical, Gyeonggi-do, South Korea) was deployed ( Fig. 4 ).

**Fig. 2:**
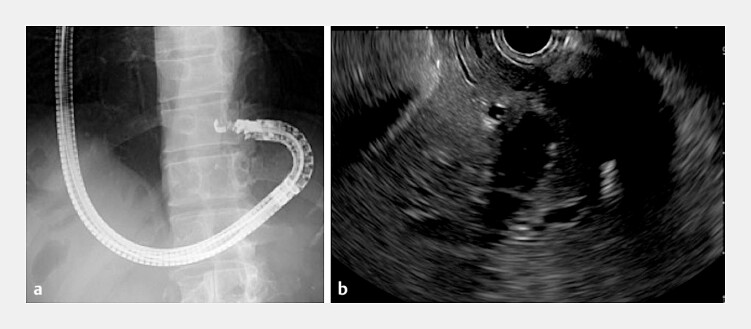
**a** A curved linear echoendoscope was advanced into the stomach following the guidewire under fluoroscopic guidance. **b** From a reversed position in the upper gastric body, a dilated intrahepatic bile duct (B2) was identified.

**Fig. 3:**
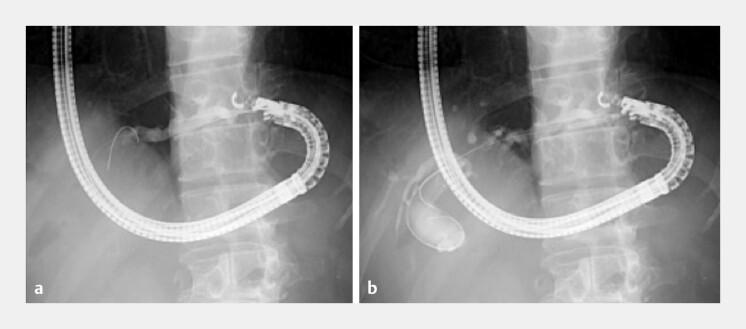
**a** Puncture was performed using a 22-G fine-needle aspiration needle, and a 0.018-inch guidewire was advanced. **b** The tract was dilated using a 4-mm balloon catheter.

**Fig. 4:**
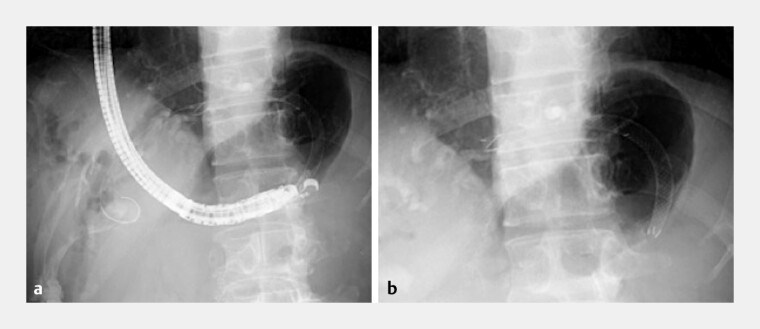
An 8 × 120-mm covered self-expandable metal stent was deployed.

Endoscopic ultrasound-guided hepaticogastrostomy in a patient with esophageal reconstruction using a free jejunal interposition graft via the presternal route.Video 1

The total procedure time was 58 minutes. There were no adverse events postoperatively, and jaundice resolved promptly. Stent dysfunction was not observed until the patient’s death from the primary disease.

EUS-HGS is a promising option for biliary drainage in patients with a malignant obstruction and complex surgical anatomy. Thorough preprocedural planning, including an understanding of the surgically altered anatomy and appropriate device selection, is essential for procedural success.

Endoscopy_UCTN_Code_TTT_1AS_2AH

## Notes

Endoscopy E-Videos https://eref.thieme.de/e-videos
*E-Videos* is an open access online section of the journal *Endoscopy* , reporting on interesting cases and new techniques in gastroenterological endoscopy. All papers include a high-quality video and are published with a Creative Commons CC-BY license. Endoscopy E-Videos qualify for HINARI discounts and waivers and eligibility is automatically checked during the submission process. We grant 100% waivers to articles whose corresponding authors are based in Group A countries and 50% waivers to those who are based in Group B countries as classified by Research4Life (see: https://www.research4life.org/access/eligibility/ ). This section has its own submission website at https://mc.manuscriptcentral.com/e-videos .
